# Turnaround times – the Achilles’ heel of community screening and testing in Cape Town, South Africa: A short report

**DOI:** 10.4102/phcfm.v12i1.2624

**Published:** 2020-10-02

**Authors:** James D. Porter, Robert Mash, Wolfgang Preiser

**Affiliations:** 1 Symphony Way CDC, Metro District Health Services, Western Cape Government, Cape Town, South Africa; 2 Division of Family Medicine and Primary Care, Department of Family and Emergency Medicine, Faculty of Medicine and Health Sciences, Stellenbosch University, Cape Town, South Africa; 3 Division of Medical Virology, Department of Pathology, Faculty of Medicine and Health Sciences, Stellenbosch University, Cape Town, South Africa

**Keywords:** COVID-19, turnaround times, community screening and testing, Cape Town, South Africa

## Abstract

Early in the course of the coronavirus infection disease 2019 (COVID-19) pandemic in South Africa, the Department of Health implemented a policy of community screening and testing (CST). This was based on a community-orientated primary care approach and was a key strategy in limiting the spread of the pandemic, but it struggled with long turnaround times (TATs) for the severe acute respiratory syndrome coronavirus-2 (SARS-CoV-2) reverse transcriptase polymerase chain reaction test. The local experience at Symphony Way Community Day Centre (Delft, Cape Town), highlighted these challenges. The first positive tests had a median TAT of 4.5 days, peaking at 29 days in mid-May 2020. Issues that contributed to long TATs were unavailability of viral transport medium, sample delivery and storage difficulties, staffing problems, scarcity of testing supplies and other samples prioritised over CST samples. At Symphony Way, many patients who tested COVID-19 positive had abandoned their self-isolation because of the delay in results. Employers were unhappy with prolonged sick leave whilst waiting for results and patients were concerned about not getting paid or job loss. The CST policy relies on a rapid TAT to be successful. Once the TAT is delayed, the process of contacting patients, and tracing and quarantining contacts becomes ineffective. With hindsight, other countries’ difficulties in upscaling testing should have served as warning. Community screening and testing was scaled back from 18 May 2020, and testing policy was changed to only include high-risk patients from 29 May 2020. The delayed TATs meant that the CST policy had no beneficial impact at local level.

## Introduction

In South Africa, the National Department of Health’s response to the growing coronavirus infection disease 2019 (COVID-19) was to introduce a policy of community screening and testing (CST). In the Cape Town Metro, it was initiated on the 6th of April 2020.^1^

Community screening and testing in Cape Town initially focused on screening in hotspot areas that were identified by the location of known cases and person under investigation (PUI) in areas with high socio-economic vulnerability.^2^ This approach built on a foundation of community orientated primary care (COPC) as it relied on the existing network of community health workers and professional nurses in the community employed by non-profit organisations and their linkages to local primary care facilities as well as various community stakeholders, such as the police. By 11th May 2020, the programme had screened 123 527 people, performed 12 330 tests, with 523 positive results (4.2%) (Personal communication, Dr Neal David).

If implemented effectively, CST should have been a key strategy to slow the spread of the severe acute respiratory syndrome coronavirus-2 (SARS-CoV-2) and allow health services to prepare for the COVID-19 peak. In the initial implementation period security concerns at mobile testing sites and the long turnaround time (TAT) for the test results were listed as significant potential problems.^2^ The ability of individuals to self-isolate or self-quarantine in vulnerable communities, where people were crowded in small houses or shacks, was also an issue and uptake of assisted isolation or quarantine was less than expected.^3^

## Local experience of community screening and testing

Our experience at Symphony Way Community Day Centre (CDC) – an 8-h, provincial primary care facility in Delft, Cape Town – highlighted the challenges of the CST policy. Three forms of screening and testing were implemented at Symphony Way CDC:

1. Mobile CST centres screened households located around known cases in the community. Community health workers (CHWs) employed by local non-profit organisations, screened household members for the recent onset (in last 14 days) of sore throat, fever, cough or shortness of breath. Those who screened positive were sent to the mobile van as PUIs and a nasopharyngeal swab was taken by staff from the CDC.
2. CHWs went into the community directly surrounding the clinic and screened community members and referred those that screened positive to the CDC for testing.
3. All patients who presented to the CDC for any reason were screened for COVID-19 and tested if they met the definition for a PUI.

## Laboratory turnaround times

**FIGURE 1:**
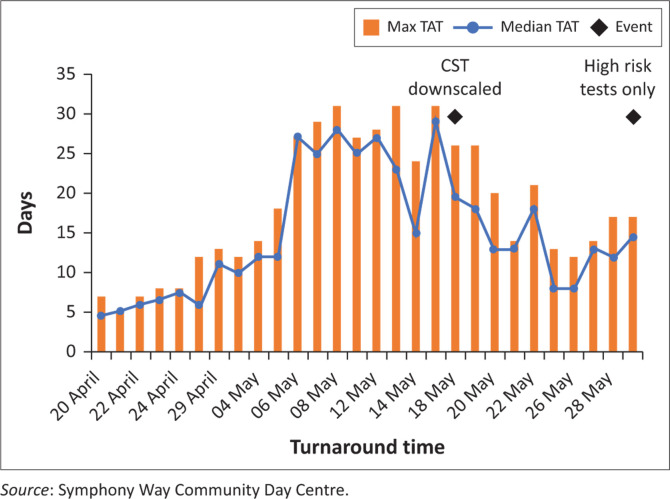
Turnaround time of positive tests for severe acute respiratory syndrome coronavirus-2 at Symphony Way Community Day Centre (*N* = 351).

There were inevitable challenges with logistics and staffing, but in general the screening and sampling process went well. However, we quickly ran into problems specifically around TATs. This was despite the National Health Laboratory Service’s (NHLS) aim of a TAT of 48 h (from date of specimen collection to date of result) for the SARS-CoV-2 reverse transcriptase polymerase chain reaction test.^4^ By early May, the TAT had become longer than the 14-day isolation period required for mild or asymptomatic cases of COVID-19.^5^ As can be seen in Figure 1, during the CST we never achieved a TAT of 48 h. We tested our first COVID-19 positive cases on the 20th of April, with a median TAT of 4.5 days. This was the quickest TAT we experienced during the whole CST campaign with TATs peaking in mid-May, when we had several positive tests with a TAT of 31 days.

A number of issues contribute to the long TATs:

1. Dry swabs were mostly used for testing as viral transport medium was largely unavailable. This added another processing step to be performed under a biosafety cabinet in the laboratory, prior to nucleic acid extraction and SARS-CoV-2 RNA amplification and detection.
2. Samples from CST were initially not delivered directly to the laboratories performing the test, but followed the routine pathways.
3. Laboratories lacked capacity in reception areas to register a high number of additional samples and were understaffed.
4. Some laboratory staff refused to deal with COVID-19 samples.
5. Some reception areas had to be shut down temporarily because of COVID-19 cases amongst staff, which was usually community acquired and staff were on sick leave because of being infected or the need to self-quarantine.
6. There was a scarcity of laboratory supplies needed for testing. The available, largely automated, high-throughput platforms suffered from a serious lack of test kits, because of insufficient production capacity, global competition and severed air cargo services. This forced laboratories to use non-automated test methods, which are labour-intensive, require specially skilled and trained staff and have low- to-medium throughput.
7. Laboratories had to prioritise the testing of samples from hospitalised and other urgent patients over samples from CST.

## Patient impact

People under investigation were meant to self-isolate at home until they received their test result. If they were negative they could de-isolate, but if they were positive then isolation would be needed for 14-days and their close contacts would also need to self-quarantine. The delay meant that many patients abandoned self-isolation when they did not receive a result within a few days and returned to work or their families. We also had many patients returning to the clinic for results, which were still pending, because of pressure from their employers. The vast majority of patients were discharged from isolation after 14-days despite not yet receiving a result and were issued with a letter for their employer explaining the situation. Many employers were not happy with this lack of clarity. Employees were concerned that they might get fired or not paid whilst they stayed at home for 14 days for a COVID-19 test result that never arrived.

## Policy impact

A rapid TAT is critical for the success of CST.^6^ The aim of CST is to slow the spread of the pandemic by finding and isolating COVID-19 positive patients and quarantining their close contacts. However, the prolonged TATs seen at Symphony Way CDC during CST meant that the majority of positive patients were contacted once they were no longer infectious, and the majority of their contacts were traced and contacted after they would already have been contagious had they also been infected. The short incubation period and possibility of pre-symptomatic infectiousness meant that many contacts would have become infectious prior to tracing even with a perfect TAT.

With the benefit of hindsight, the experiences of other countries that struggled to ramp up laboratory testing in response to the pandemic spread (such as UK and USA) should have served as a warning. To achieve the aspired slowing of community spread, the CST strategy should have been centred around the use of near-patient testing. Ironically, South Africa has a large network of GeneXpert machines for near-patient tuberculosis diagnosis,^7^ despite their suboptimal integration into the healthcare process^6^ and availability of an assay for SARS-CoV-2,^8^ which in theory would have allowed for this; yet massively insufficient supplies of test cartridges made this option not feasible. As in so many other areas, the pandemic has revealed pre-existing shortcomings and failures.

The rapidly rising TATs prompted our substructure to start scaling back on CST with the mobile testing centres no longer operational from 18 May 2020. The policy of testing all PUIs in the Cape Town Metro was eventually stopped on 29 May 2020. A new policy was introduced, which meant that only those older than 55 years or with comorbidity were investigated in public sector primary care.^9^ This change in testing strategy was dictated by the circumstances surrounding prolonged TATs rather than the goals of CST. It has effectively moved the approach of the Cape Town Metro from preventing local transmission to case finding in high-risk people.

## Conclusion

Our experience of the CST policy at Symphony Way CDC in Delft, Cape Town, was unfortunately a negative one. Although the rationale behind CST was sound, the simple fact that TATs were so delayed meant that it was never as effective as hoped. Despite the efforts of all healthcare workers involved, the contacting and tracing process was always too delayed to have any beneficial impact at a local level. It is likely that the national lockdown rather than CST was responsible for slowing spread of the infection. Given the lack of rapid testing, perhaps a targeted approach should have been implemented from the beginning. Further evidence on appropriate or alternative approaches to CST in low or middle income countries settings with low laboratory capacity must still be collected.
